# Disorders of Consciousness, Language and Communication Following Severe Brain Injury

**DOI:** 10.5334/pb.1381

**Published:** 2025-06-11

**Authors:** Charlène Aubinet, Anaïs Gillet, Amandine Regnier

**Affiliations:** 1 Coma Science Group, GIGA-Consciousness, GIGA Institute, University of Liège, Belgium; 2 NeuroRehab & Consciousness Clinic, Neurology Department, University Hospital of Liège, Belgium; 3 Psychology and Neuroscience of Cognition Research Unit, University of Liège, Belgium

**Keywords:** Disorders of consciousness, Language, Communication, Neurorehabilitation, Aphasia, Coma

## Abstract

Patients with severe brain injuries and disorders of consciousness (DoC) represent a complex clinical population in terms of diagnosis, prognosis, and management, including critical ethical considerations. Behavioral assessment scales remain the primary tools for evaluating the level of consciousness of these patients following a coma; however, they heavily depend on language and communication abilities. This reliance can lead to underestimating residual consciousness in cases where language impairments go undetected. Accordingly, the latest international guidelines on DoC diagnosis have highlighted aphasia as a significant confounding factor that must be addressed. On the other hand, accurately assessing residual language abilities is essential for better characterizing the patient’s cognitive profile. This, in turn, enables neuropsychologists and speech-language therapists to tailor and plan effective rehabilitation programs. This review examines the current literature on language function and communication skills in patients with DoC, detailing the latest tools for assessing and managing language and consciousness in individuals with severe brain injuries. We explore the critical role of language function in evaluating residual consciousness, particularly in DoC behavioral diagnoses and in identifying covert consciousness through neuroimaging passive or active paradigms. Furthermore, we discuss how therapies aimed at recovering consciousness—such as pharmacological treatments, electromagnetic therapies, sensory or cognitive stimulation, and communication aids like brain-computer interfaces—may also impact or rely on language function and communication abilities. Further research is needed to refine methodologies and better understand the interplay between language processing, communication and levels of consciousness.

## 1. Introduction

Over the past fifty years, advancements in mechanical ventilation and medical care have significantly improved the prognosis of patients with brain injuries. The increase in survival rates has, however, multiplied cases of complex and prolonged disorders of consciousness (DoC). DoC are characterized by the alteration of one or more components of consciousness, including the level of arousal, self-awareness, and awareness of the environment (Martial et al., 2020).

**Figure 1:**
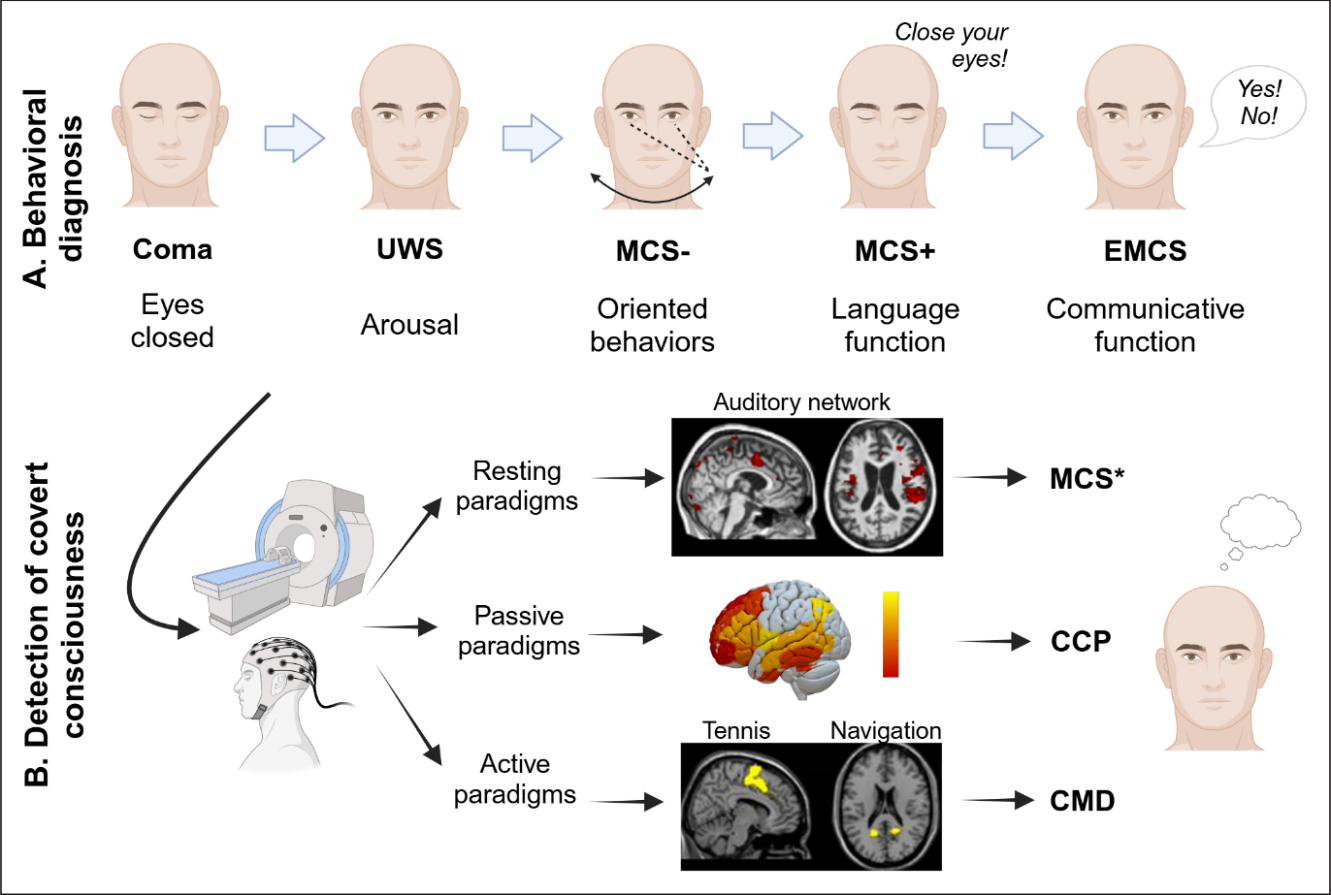
**Characterization of consciousness levels after severe brain injury. A**. Diagnosis is based on behavioral assessments: unresponsive wakefulness syndrome (UWS), minimally conscious state (MCS) and emergence from the MCS (EMCS). **B**. Neuroimaging or electrophysiological paradigms detect covert consciousness: non-behavioral MCS (MCS*) is identified in the resting state, covert cortical processing (CCP) in passive listening tasks, and cognitive motor dissociation (CMD) in active mental imagery tasks. Created with Biorender.com.

At the behavioral level (Figure 1A), coma is first described as constant eye closure lasting from a few hours to up to four weeks (except pharmacological coma which may last longer) (Sanz et al., 2018). The Unresponsive Wakefulness Syndrome (UWS; also called vegetative state) is then defined by the recovery of arousal and includes preserved eye opening and autonomic functions, but without goal-directed movements or language (Sanz et al., 2018). Patients who recover such fluctuating signs of consciousness are considered as in a Minimally Conscious State (MCS) (Giacino et al., 2002), which is divided into two subtypes: the MCS *minus* (MCS-; without evidence of language function) and the MCS *plus* (MCS+; with evidence of preserved language function) (Bruno et al., 2011; Thibaut et al., 2020). The emergence from the MCS (EMCS), defined by consistent and functional object use and/or communication, is finally associated with full consciousness recovery and thus not considered a DoC (Sanz et al., 2018). The Locked-In Syndrome (LIS) is also not included in the DoC taxonomy as patients retain cognitive functions, communicative abilities and awareness, but are unable to perform motor movements, except eye or distal movements in certain cases (Sanz et al., 2018).

In the last decade, novel DoC labels additionally emerged based on patients’ neural responses in neuroimaging and electrophysiological paradigms. The term Non-Behavioral MCS or MCS* was first proposed in 2014 by Gosseries and colleagues, to characterize patients who show no conscious behaviors (i.e., UWS), but residual brain activity compatible with the diagnosis of MCS (Gosseries et al., 2014). More specifically, behaviorally unresponsive patients may show appropriate neural responses either in active tasks (e.g., mental motor imagery) – known as a Cognitive Motor Dissociation (CMD) (Bodien et al., 2024; Schiff, 2015) – or in passive tasks (e.g., tactile or auditory stimulation) – called Covert Cortical Processing (CCP) (Edlow et al., 2021; Young, Fecchio, et al., 2024). These different categories of covert consciousness are illustrated in Figure 1B.

An accurate diagnosis of these states is crucial since it has important implications in terms of prognosis (Estraneo et al., 2020), treatment (Thibaut, Schiff, et al., 2019), pain management (Chatelle et al., 2016) and ethical considerations including end-of-life decisions (Demertzi et al., 2011). Bedside standardized behavioral assessment scales are the first-line assessments for practicability, cost and accessibility reasons (Schnakers & Majerus, 2011; Thibaut et al., 2021). Nevertheless, the reliance on language and communication abilities in these scales presents a challenge, as unrecognized language difficulties can lead to an underestimation of a patient’s consciousness level (Aubinet, Schnakers, et al., 2022; Majerus et al., 2009). The most recent international guidelines on DoC diagnosis indeed recognized the presence of aphasia as an important confounding factor that should be taken into consideration (Giacino et al., 2018; Kondziella et al., 2020). On the other hand, an accurate assessment of residual linguistic abilities leads to a better characterization of the patient’s cognitive profile, which in turn helps neuropsychologists and speech-language therapists orient and plan their rehabilitation programs (Aubinet, Murphy, et al., 2018; Aubinet, Schnakers, et al., 2022).

The aim of this review is to consolidate current knowledge about language and communication abilities in patients with DoC. We will first describe the role of language function in diagnosing these conditions based on both behavioral tools and neuroimaging techniques. We will furthermore discuss current interventions aimed at managing language and communication in these patients with impaired consciousness levels, including cognitive stimulation and communication aids.

## 2. Language, Communication and Behavioral Diagnosis of Consciousness

### 2.1. Behavioral scales assessing consciousness

As soon as patients open their eyes in the intensive care units, clinicians regularly use verbal commands such as ‘squeeze my hand’ to probe for consciousness. Compared to clinical consensus, the use of standardized behavioral scales importantly reduces the rate of DoC misdiagnosis (Schnakers et al., 2009). Commonly used scales include the well-known Glasgow Coma Scale (GCS), which assesses visual, motor, and verbal responses in acute conditions (Teasdale & Jennett, 1974), or the Wessex Head Injury Matrix (WHIM), which can also be used in chronic settings to follow the recovery of communication, attention, social behavior, concentration, visual awareness and cognition (Majerus et al., 2000; Shiel et al., 2000). Moreover, the Coma Recovery Scale-Revised (CRS-R) examines arousal, auditory and visual perception, motor and oro-motor abilities as well as communication skills (Giacino et al., 2004). The use of this scale is currently recommended as it is the most sensitive tool to differentiate between UWS and MCS patients (Seel et al., 2010). Yet, the CRS-R requires extensive training and is time-consuming to administer, especially since repeated assessments are needed to reduce misdiagnosis (Wannez et al., 2017).

More recently, the Simplified Evaluation of CONsciousness Disorders (SECONDs) was developed to provide a fast and easy-to-use scale to diagnose consciousness levels (Aubinet, Cassol, et al., 2021; Sanz et al., 2021). This scale includes the five most frequent signs of MCS (i.e., visual fixation, visual pursuit, oriented behaviors, pain localization and command-following) (Wannez et al., 2017), as well as arousal and communication items. Given its substantial to almost perfect agreement with DoC diagnosis using the CRS-R (κ = 0.78 and 0.85), the SECONDs tool is a good alternative in clinical settings where time constraints prevent a more thorough assessment. In the same vein, the Coma Recovery Scale-Revised For Accelerated Standardized Testing (CRSR-FAST) facilitates serial assessment of consciousness in acute settings, by including only those CRS-R behaviors that differentiate conscious (i.e., MCS or EMCS) from unconscious (i.e., coma or UWS) patients (Bodien et al., 2023). This practical and valid assessment scale therefore improves consciousness detection and prognostic accuracy in the intensive care units.

Overall, the current literature on DoC describes a variety of behavioral scales for diagnosing acute and chronic DoC. Nevertheless, all of them crucially include verbal instructions that patients need to understand to perform the items appropriately. As described in the next section, language abilities and consciousness levels are indeed closely intertwined.

### 2.2. Conscious behaviors relying on language function

**Figure 2:**
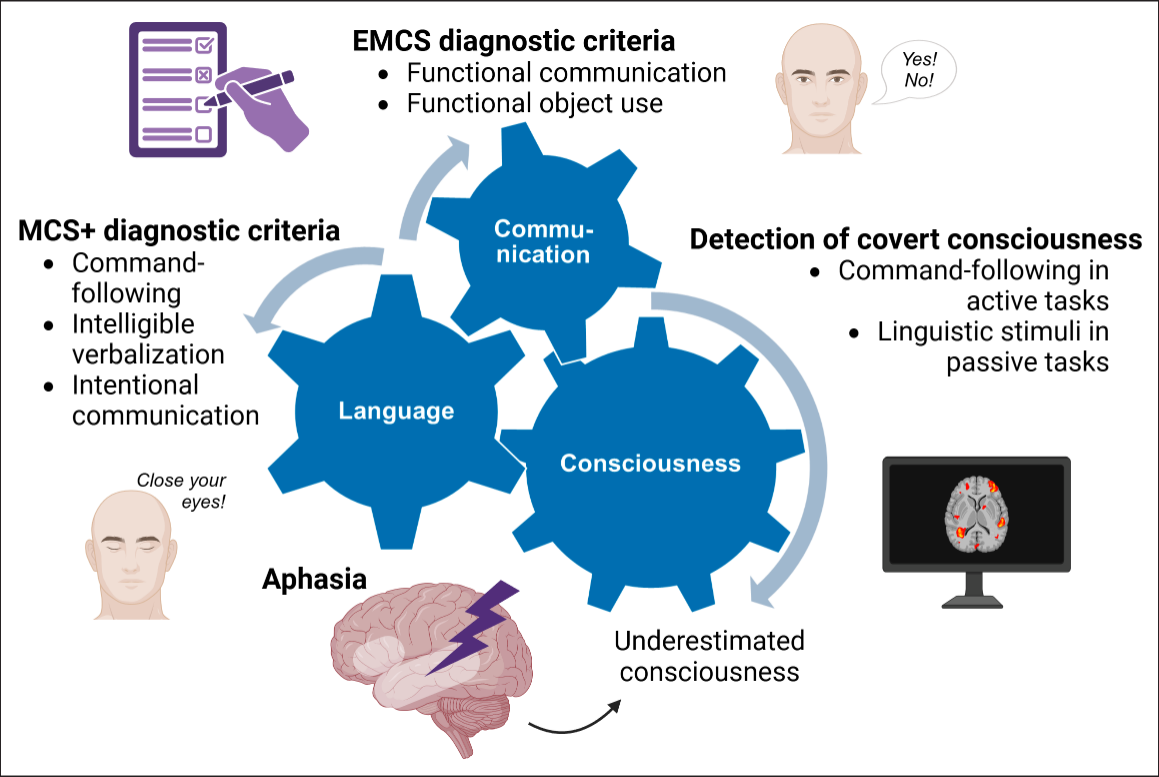
**Interconnection of language, communication, and consciousness**. Language-related and communication items are included in standardized behavioral assessment scales as criteria for MCS+ and EMCS diagnoses. Neuroimaging or electrophysiological passive and active paradigms detect residual covert consciousness based on language stimuli. Consequently, language impairments may lead to an underestimation of patients’ consciousness level. Created with Biorender.com.

As illustrated in Figure 2, language and communication are fundamental abilities when considering the diagnosis of DoC. The current taxonomy first differentiates between MCS- and MCS+ based on the absence or presence of residual language function (Bruno et al., 2011). The MCS+ syndrome is indeed diagnosed based on command-following (i.e., relying on language comprehension), intelligible verbalization or intentional communication capacities (Thibaut, Bodien, et al., 2019).

In line with this, several neuroimaging studies confirmed that MCS+ patients exhibit higher brain activity in the language network compared to MCS- patients (Aubinet, Murphy, et al., 2018; Aubinet et al., 2019, 2020; Bruno et al., 2012; Ferraro et al., 2020). Although no differences in gray matter volume were found between MCS- and MCS+ clinical groups, higher metabolism in regions related to semantic processing (i.e., left middle temporal cortex) and preserved connectivity within the left frontoparietal network were observed in MCS+ compared to MCS- patients (Aubinet et al., 2020). Using resting-state functional magnetic resonance imaging (fMRI), higher functional connectivity in the left frontoparietal language network was also described in the MCS+ compared to the MCS- group. However, no significant differences were found in the thalamocortical network (i.e., related to internetwork information integration), the right frontoparietal network (i.e., related to the perception of surroundings), or the default mode network (i.e., related to internal awareness and self-mentation) (Aubinet, Larroque, et al., 2018). According to this research, the MCS subgroups actually show neural activity differences in the language network, rather than in regions classically associated to consciousness, such as the thalamus or the posterior cingulate cortex.

Moreover, the recovery of command-following and language function more broadly sustain the reappearance of communication, which is a key behavioral sign that a patient is progressing toward consciousness (Murtaugh et al., 2024). According to the CRS-R, the specific criterion for functional communication is 6/6 on situational yes/no questions, which can be auditory (e.g., ‘Am I clapping my hand right now?’) or visually based (e.g., ‘Am I touching my ear right now?’) (Giacino et al., 2004). Consistent and appropriate responses lead to the diagnosis of EMCS.

Consequently, language impairment is known to interfere with the assessment of consciousness (Majerus et al., 2009). The extent of brain lesions in DoC patients may cover the language network, involving a high probability of language impairment (i.e., aphasia) in this population. As command-following and communication items are commonly failed in aphasic patients, they are at high risk of consciousness misdiagnosis. This was particularly highlighted in a previous study, where half of fully conscious patients with global aphasia were misdiagnosed as in a MCS when performing the CRS-R assessment (Schnakers et al., 2015). For example, patients with severe language impairments may not be able to achieve the accuracy of response required for yes/no communication tasks.

In brief, potential concomitant language deficits in DoC patients are considered as important confounding factors in the assessment of consciousness levels after coma (Diserens et al., 2023; Pundole & Crawford, 2018). In this context, the current recommendation is to first assess the level of consciousness in patients using behavioral scales, followed by a more specific evaluation of residual language abilities through dedicated assessment tools (Aubinet, Schnakers, et al., 2022).

### 2.3. Specific language and communication assessments

**Figure 3:**
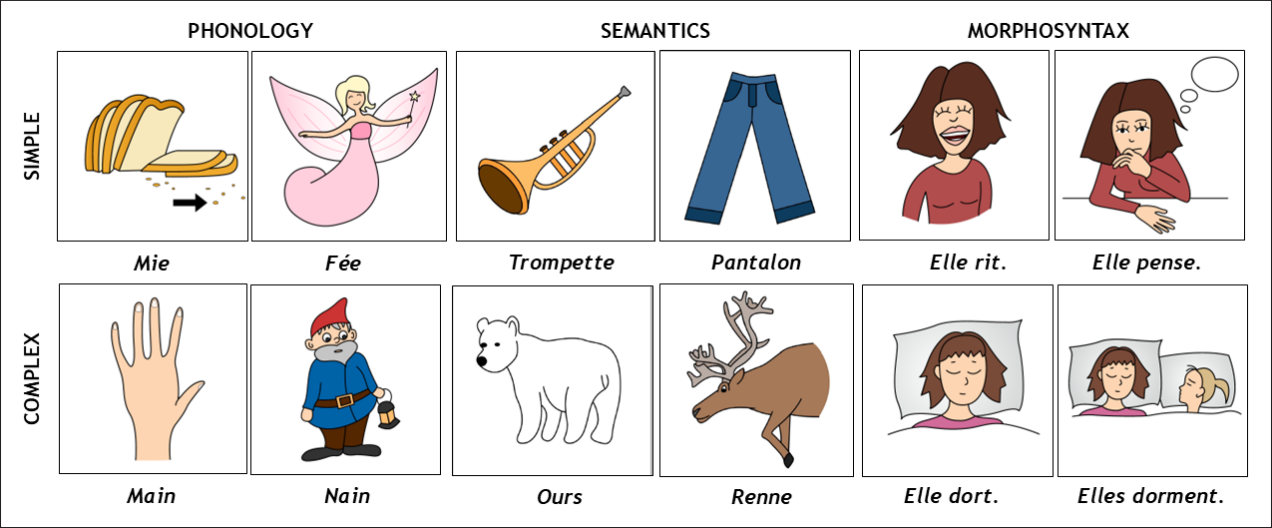
**Examples of items from the French version of the Brief Evaluation of Receptive Aphasia tool**. Simple phonological items require the discrimination of monosyllabic words that do not share any phonemes, while complex phonological items involve minimal pairs (i.e., monosyllabic words differing by only one of two phonemes). Simple semantic items involve distinguishing words from two different semantic categories, whereas complex semantic items contrast words belonging to the same semantic category. This semantic subscale also controls for word frequency. Finally, simple morphosyntactic items consist of two sentences with different meanings but sharing the same morphosyntactic structure, while complex morphosyntactic items contrast sentences that differ across multiple morphosyntactic features (e.g., number, gender, active vs. passive voice…).

A novel tool, the Brief Evaluation of Receptive Aphasia (BERA), has been developed to specifically assess residual language comprehension in this challenging population. This tool is composed of two versions of 30 items, allocated to phonological, semantic, and morphosyntactic subscales. Examples of items are illustrated in Figure 3. Concretely, patients are required to look at a picture corresponding to the word or sentence pronounced by the examiner, which is presented next to a specific distractor to control for different psycholinguistic variables. As a first step, the BERA tool was administered in 52 aphasic conscious patients to determine its sensibility to language impairment (Aubinet, Chatelle, et al., 2021). In this aphasic group, the BERA showed very good psychometric properties (i.e., intra- and inter-rater reliability, content and concurrent validity). This tool was furthermore administered to four MCS or EMCS patients with visual fixation and pursuit (i.e., prerequisites for performing this scale) to demonstrate the feasibility of this assessment in post-comatose patients. Their BERA scores suggested the presence of specific receptive difficulties for phonological, semantic and particularly morphosyntactic subscales, and their results were in line with functional and structural neuroimaging data (Aubinet, Chatelle, et al., 2021). The French version of the BERA assessment is currently being validated in a larger patient group with promising preliminary results (Aubinet et al., 2024), but it has also recently been translated and adapted to English and various other languages. A computerized BERA tool using an eye-tracking device is finally being developed to provide more objective measures of patients’ eye movements.

The assessment of communication abilities is mostly considered based on the establishment of a yes/no code in standardized behavioral scales. If patients are unable to pronounce the words ‘yes’ and ‘no’, they also need to learn coded yes/no responses and recall them (e.g., ‘yes’ with eyes up and ‘no’ with eyes down, eye pointing to written yes/no cards…) (Pundole et al., 2021). This makes the task even more challenging, as it requires not only understanding the question but also recalling and applying the code while simultaneously holding both the question and response in working memory. Even patients who have clearly regained consciousness after traumatic brain injury may find situational and judgement questions difficult to answer (Nakase-Richardson et al., 2009). A lack of confidence in such communication items was therefore reported in almost half of clinicians working in specialist rehabilitation settings (Pundole et al., 2021). These authors stressed the need for updated recommendations on the tasks for determining the return of functional communication.

The use of the Functional Communication Measures (FCM) was recently suggested to specifically evaluate cognitive requirements for communication and communicative abilities in post-comatose patients (De Bellis et al., 2022). The FCM consists of a series of rating scales, ranging from 1 (least functional) to 7 (most functional), assessing attention, memory, swallowing, communicative-augmentative communication, motor speech, spoken language expression, and spoken language comprehension. An Italian adaptation of the FCM was administered to two EMCS patients and identified two different functioning profiles in the attention, swallowing, motor speech, and spoken language expression scales, even though these two patients achieved the same scores on scales for functional disability and consciousness level (De Bellis et al., 2022). The FCM might therefore be a promising and easy-to-administer tool to assess communicative functions in these patients with severe brain injury.

To conclude this section, the behavioral diagnosis of DoC remains challenging due to the complex interplay between residual consciousness, language impairments, and behavioral limitations (Figure 2). While assessment scales such as the CRS-R and SECONDs provide critical tools for differentiating consciousness levels, their reliance on language and communication abilities poses significant challenges, especially in patients with aphasia. Emerging tools like the BERA and FCM offer promising avenues for assessing residual linguistic and communicative capacities, thereby improving diagnostic precision and tailoring rehabilitation efforts. Along with such bedside assessments, the use of electroencephalography (EEG) and fMRI is particularly suggested to circumvent language or motor impairments and improve diagnostic accuracy of DoC patients (Alnagger et al., 2023; Majerus et al., 2009). Although some patients may not exhibit overt signs of consciousness at the bedside, covert consciousness can indeed be identified through diverse techniques (Schiff, 2015). The next section will focus on these neuroimaging paradigms.

## 3. Language and Detection of Covert Consciousness

Multimodal assessments have been recommended by both the American and European Academies of Neurology in the case that repeated behavioral assessments in patients with DoC result in ambiguous conclusions (Giacino et al., 2018; Kondziella et al., 2020). Neuroimaging and electrophysiological techniques have indeed revealed vast insights into the relationships between neural impairments and behavioral signs of consciousness in DoC, which have led to the development of specific paradigms for the clinical assessment of patients with severe brain injury (Alnagger et al., 2023). Table 1 summarizes the techniques and paradigms that highlight key indicators suggestive of covert consciousness.

**Table 1:** Neuroimaging and electrophysiological signs suggestive of covert consciousness.


	RESTING BRAIN ACTIVITY	PASSIVE PARADIGMS	ACTIVE PARADIGMS

FDG-PET	Preservation of brain metabolism in the frontoparietal cortex (Thibaut etal., 2021)	Preserved association cortices activation to patients’ own name (Laureys et al., 2004)	/

fMRI	Preserved functional connectivity in auditory and default mode networks (Demertzi et al., 2015)	Preserved brain activity associated with higher-order processing of linguistic stimuli, including activation in the temporal lobe (Coleman et al., 2009)	Brain activation consistent with command-following in motor imagery or silent naming tasks (Owen et al., 2006; Rodriguez Moreno et al., 2010)

EEG	Global functional connectivity and power spectrum preservation (Duszyk-Bogorodzka et al., 2022; Thibaut et al., 2021)	Preserved event-related potentials (e.g., N400 wave) to linguistic stimuli(Balconi et al., 2013; Balconi & Arangio, 2015; Formisano et al., 2019) or speech-tracking responses (Braiman et al., 2018; Gui et al., 2020; Sokoliuk et al., 2020)	Command-following capacity reflected by power spectrum changes during motor imagery (Claassen et al., 2019) or preserved event-related potentials (e.g., P300) during counting tasks (Guger et al., 2018)


### 3.1. Resting brain activity

Several techniques can be employed to quantify spontaneous brain activity in resting state conditions, during which the patients are not performing any task. These resting state paradigms are ideal for clinical use, particularly as they are easy to perform and do not require any collaboration from the patients.

Using resting-state fluorodeoxyglucose positron emission tomography (FDG-PET), brain metabolism in the frontoparietal cortex shows high sensitivity and specificity in differentiating UWS from MCS patients (Stender et al., 2014). Combining appropriate and repeated behavioral assessments with FDG-PET, Thibaut and colleagues showed that the proportion of ‘true’ UWS patients (i.e., behaviorally unresponsive patients with frontoparietal hypometabolism) in the whole DoC population was rather small (12%) (Thibaut et al., 2020). In this study, patients in MCS* (i.e., behaviorally unresponsive patients with preserved frontoparietal metabolism) had better outcomes than UWS patients and exhibited grey matter structure and global functional connectivity (using high-density EEG) that are more compatible with MCS patients than UWS patients.

Neural correlates of consciousness were additionally highlighted based on Blood Oxygenation Level Dependent (BOLD) signals in resting state fMRI (Demertzi et al., 2015; Threlkeld et al., 2018; Vanhaudenhuyse et al., 2011). Functional connectivity was investigated for the frontoparietal, salience, auditory, sensorimotor, visual and default mode networks, all of them showing high discriminative capacity (>80%) for separating patients in MCS and UWS (Demertzi et al., 2015). The auditory network showed the best capacity to discriminate between both groups, whereas other studies observed a preserved default mode network in MCS compared to UWS patients (Threlkeld et al., 2018; Vanhaudenhuyse et al., 2011). Default mode network properties in patients with evidence of covert consciousness are however more similar to patients with a behavioral diagnosis of coma, UWS, and MCS- than to overtly conscious patients (Bodien et al., 2019). Further research is therefore needed to clarify whether resting state fMRI could be related to the presence of covert consciousness in DoC patients.

Finally, resting state EEG is commonly used to assess brain activity profiles associated with the presence or absence of preserved consciousness (Duszyk-Bogorodzka et al., 2022). EEG methods offer several advantages, such as portability, low cost, easy bedside application, and high temporal resolution. However, the wide range of analytical approaches poses a challenge for methodological standardization (Schnakers et al., 2020). In UWS patients, brain activity is characterized by the dominance of low-frequency oscillations, primarily in the delta range, and a reduction in higher frequencies—both in terms of global power and functional connectivity patterns. In contrast, MCS patients exhibit preserved higher-frequency activity, both in global power and in long-distance functional connections (Duszyk-Bogorodzka et al., 2022). A recent study also particularly highlighted the potential utility of restored alpha networks as a biomarker to detect covert cognition in DoC (Zhou et al., 2024).

In any event, resting state approaches imply that consciousness cannot be directly inferred, providing measures of capacity for consciousness at the most (Young, Edlow, et al., 2024). In this context, passive and active paradigms are increasingly used to detect residual brain activity reflecting conscious cognition after coma (Bodien et al., 2024; Edlow & Menon, 2024). As described in the next two sections, both paradigms importantly involve residual language processing.

### 3.2. Language in passive paradigms

Different passive listening paradigms can be used to detect covert cortical processing through fMRI techniques (Cherkasova et al., 2023). A first approach involved presenting patients with noise, intelligible and unintelligible speech (Coleman et al., 2007, 2009; Schiff et al., 2005). Notably, two patients clinically diagnosed as being in an UWS demonstrated some brain activity associated with high-order processing, including temporal lobe activation (Coleman et al., 2009). Furthermore, this study demonstrated that patients exhibiting such processing had a better recovery prognosis within six months following the assessment, which supported the idea that covert consciousness in patients implies a better prognosis.

Alternatively, significant fMRI brain responses to factually incorrect compared to correct sentences were found in UWS and MCS patients, and mainly recorded in left-sided language-related areas such as Broca and Wernicke areas (Kotchoubey, Yu, et al., 2013). More recently, Nigri and colleagues examined different language effects by contrasting fMRI responses to pairs of words and pseudowords (Nigri et al., 2017). Activations in language areas reflecting low-level auditory, pseudoword, lexical or semantic relatedness effects were observed, including in some UWS patients, although they were less extended and spottier than in healthy subjects. Another interesting study suggested the use of specific narratives in an inventive passive paradigm including ‘DoC+’ (i.e., covertly aware) and ‘DoC-’ (i.e., with no covert or overt evidence of command-following) patients (Naci et al., 2018). During the broadcast of a plot-driven narrative from the kidnapping scene of the movie “Taken” (i.e., suspenseful audio), the DoC+ group showed down-regulation of the connectivity of auditory and frontoparietal (i.e., auditory-dorsal attention and executive control) networks and significantly differed from the DoC- group who did not show this effect. The functional differentiation between the auditory and frontoparietal systems also decreased significantly relatively to the level of consciousness. Interestingly, stronger functional differentiation between these systems in response to speech stimulation finally predicted higher intellectual abilities in healthy subjects during conscious cognition.

Over the years, various passive paradigms based on EEG have furthermore been developed to complement the behavioral assessment of DoC. Some of them record electrical signals in response to perturbation of the brain, such as that caused by auditory stimulation (i.e., event-related potentials; ERPs), peripheral electrical or tactile stimulation (e.g., somatosensory evoked potentials), or direct electrical or magnetic stimulation of the brain (e.g., transcranial magnetic stimulation) (Edlow et al., 2021). Responses to linguistic stimuli were particularly highlighted as markers of higher-order processing with prognostic value. For example, negative ERPs at 400 ms (i.e., N400 effect) associated with semantic processing can be observed in response to incongruous sentence endings, with responses that are delayed in unresponsive patients (Balconi et al., 2013; Balconi & Arangio, 2015). In a more recent study, the N400 component was detected in 64% of acute DoC patients (Formisano et al., 2019). This effect significantly predicted their recovery, while the absence of N400 was significantly associated with the presence of aphasia diagnosed at the clinical follow-up when patients were EMCS. Additionally, the neural activity tracking speech signal over time (i.e., speech envelope) can be evaluated by correlating the EEG responses with speech features. The natural speech envelope may stratify patients with severe brain injuries and help identify CMD patients (Braiman et al., 2018). Moreover, Gui and colleagues showed that EEG-derived neural signals of language processing, including both speech-tracking responses and temporal dynamics of global brain states, can be associated with the type of behavioral DoC (Gui et al., 2020). Phrase- and sentence-level responses decreased in MCS (relative to healthy controls) and fully disappeared in UWS. According to their results, as language stimuli become more complex (i.e., from words to entire sentences), the associated neural signals become more reliable in differentiating UWS from MCS patients. Another example comes from Sokoliuk and colleagues, who recorded EEG brain activity in unresponsive acute patients who were hearing streams of monosyllabic words that built meaningful phrases and sentences (Sokoliuk et al., 2020). The results showed a significant correlation between functional outcomes at 3- and 6-months and the strength of patients’ acute cortical tracking of phrases and sentences, quantified by inter-trial phase coherence. EEG data can finally be recorded while patients listen to a suspenseful audio (Laforge et al., 2020). This approach has revealed patterns of activity like those observed in healthy participants in 38% of patients, including four patients diagnosed with UWS. Specifically, these patterns included widespread frontal negativity and generalized posterior positivity.

Comparing EEG and fMRI approaches, another study proposed some tasks to detect covert consciousness in intensive care unit patients (Edlow et al., 2017). The language paradigm involved presenting excerpts of speech played forwards and backwards to identify differences in the processing of linguistic and non-linguistic aspects of speech. Their findings indicated that patients who demonstrated language abilities at the bedside (i.e., MCS+ and EMCS) were more likely to respond to both language and music paradigms than to mental imagery tasks. However, fMRI examination did not reveal activity like that observed in control participants for three of their patients with preserved language abilities confirmed at the bedside. Furthermore, EEG examination demonstrated an important lack of sensitivity as it failed to detect language-specific activity in two healthy participants and in two patients with preserved bedside-assessed language abilities. This study underscores the need to refine paradigms and methodologies to achieve tests that can reliably and consistently reveal preserved language abilities. The same authors recently defined the CCP as the detection of association cortex responses to language (and music) in patients who do not show evidence of language function (e.g., command-following capacity) on behavioral assessments (Edlow et al., 2021).

In a previous review (Aubinet, Chatelle, et al., 2022), we showed that the overall rate of patients showing sensitivity to language stimuli increases from low (i.e., UWS) to high levels of consciousness (i.e., MCS and EMCS). One third of UWS patients would present a CCP, although their brain response to linguistic stimuli are slower, weaker and spatially less extended compared to MCS patients (Aubinet, Chatelle, et al., 2022). Overall, levels of language processing and consciousness may correlate after severe brain injury. More studies will however be needed to better characterize how language comprehension and consciousness recovery may interact with each other.

### 3.3. Language in active paradigms

Numerous studies have shown that some patients with UWS exhibit brain activation when exposed to verbal commands, even if no behavioral response is detected (Bodien et al., 2024; Claassen et al., 2019; Kondziella et al., 2016). These tasks involving linguistic processing and volitional control appear to offer the greatest prognostic value (Aubinet, Schnakers, et al., 2022). This is in line with behavioral data showing that MCS+ patients with residual language abilities have a better chance of recovering functional behaviors and exhibit reduced disability after six weeks of follow-up, compared to MCS- or UWS patients (Giacino et al., 2020; Llorens et al., 2024).

Active tasks involve language comprehension by using verbal instructions, where patients can be asked, for example, to imagine themselves playing tennis or opening and closing their hand (Claassen et al., 2019; Monti et al., 2010; Owen et al., 2006). For example, Owen and colleagues used fMRI in a behaviorally unresponsive patient to show appropriate activation to motor imagery (i.e., when imagining playing a game of tennis) in the supplementary motor area, whereas activation to spatial imagery (i.e., when imagining visiting all of the rooms of her house) was found in the parahippocampal gyrus, posterior parietal lobe and lateral premotor cortex (Owen et al., 2006). Another EEG approach involves attempting to elicit a P300 wave by asking patients to count the number of rare tactile stimulations on either the right or left hand to establish a yes/no communication code (Guger et al., 2018; Spataro et al., 2022). Specifically, the patient is instructed to count the tactile stimulations on the target wrist (e.g., left wrist for ‘yes’ and right wrist for ‘no’). The system then detects cerebral responses via EEG by analyzing the difference between target and non-target stimulations. This paradigm allowed the authors to detect covert consciousness in 41% of their patients, all behaviorally diagnosed as UWS (Guger et al., 2018). Alternatively, patients can also be asked to silently name objects presented on the screen (Rodriguez Moreno et al., 2010) or to follow logical and grammatical statements (e.g., ‘The house precedes the face’), during which they must imagine which object is in front (Hampshire et al., 2013).

Next to these EEG and fMRI approaches, another promising neuroimaging technique used to detect covert command-following abilities is the functional near-infrared spectroscopy (fNIRS), a non-invasive method that measures brain activity by monitoring changes in blood oxygenation (Kazazian et al., 2024; Nagels-Coune et al., 2021). It uses near-infrared light, which is emitted through the skull and absorbed differently by oxyhemoglobin (HbO) and deoxyhemoglobin (HbR), then measures the amount of reflected light to infer changes in blood oxygenation. Compared to fMRI, this technique is portable, relatively affordable and easier to operate (Nagels-Coune et al., 2021). Following the mental imagery paradigm described by Owen and colleagues (Owen et al., 2006), this technique enabled to detect cortical responses at the bedside of patients in intensive care units (Bicciato et al., 2022; Kazazian et al., 2024).

Recently, a large-scale study using EEG and fMRI techniques involved 353 patients from 6 different centers, including coma, UWS and MCS- patients, and revealed a rate of 25% of patients with CMD (Bodien et al., 2024). Another interesting result of this study is that 62% of patients who were able to respond to the command during behavioral assessments were unable to perform the cognitive task. This demonstrates that the neuroimaging techniques used to detect CMD can have a high false-negative rate, suggesting that the actual rate of CMD patients may be even higher than the 25% observed in this study. Short-term memory issues, fatigue and the quality of the methods used, are some examples of factors that may hinder the observation of this type of residual cognition (Kotchoubey, Veser, et al., 2013; Porcaro et al., 2022).

Even more importantly, significant language comprehension difficulties may again crucially interfere with the detection of residual consciousness (Murtaugh et al., 2024). A recent study showed the impact of aphasia on brain activation to verbal motor commands in patients in acute intracerebral hemorrhage (Jacobson et al., 2024). These patients had behavioral evidence of command-following or were able to mimic motor commands, and they underwent an EEG-based motor command paradigm used to detect CMD as well as comprehensive aphasia assessments. The results showed that brain activation to motor commands is four times less likely for patients with impaired comprehension (i.e., receptive or global aphasia). This study again stressed the need to consider the presence of aphasia in consciousness assessments, when interpreting CMD testing in patients with DoC.

In sum, the detection of covert consciousness in patients with DoC relies on a combination of approaches, including resting-state brain activity analyses and passive and active stimulus-response paradigms, in line with the crucial role of language processing (Figure 2). In the next section, we will discuss different therapeutic interventions that may contribute to consciousness, language and communication recovery in patients with DoC. Among them, active paradigms are used as Brain-Computer Interfaces (BCIs) that could be adapted to individual patients and included in interventions for managing language and communication in patients with DoC.

## 4. Language and Communication Therapies in Disorders of Consciousness

Neuropsychologists and speech-language therapists may be part of interdisciplinary care teams, which also include psychologists, physiatrists, neurologists, nurses, physical therapists, occupational therapists or social workers, and contribute to supporting the recovery process for patients with DoC (Giacino et al., 2018). Rehabilitation of patients with severe brain injury and DoC is an ongoing process that requires continuous and multimodal assessment (Kondziella et al., 2020) and adaptation of strategies based on individual needs and progress.

**Figure 4:**
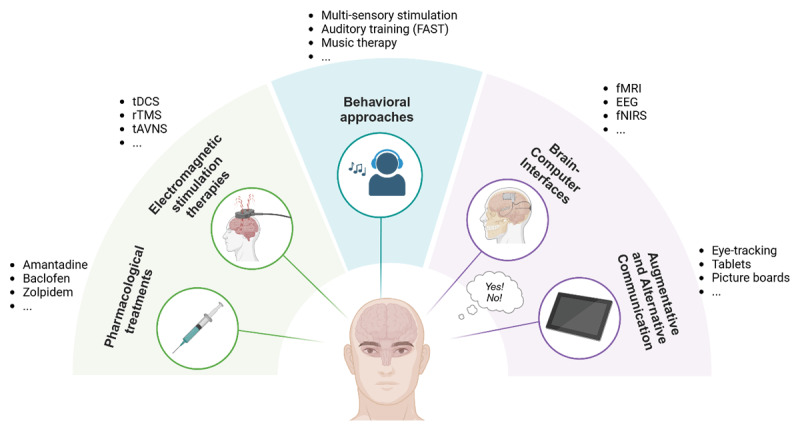
**Overview of current interventions for consciousness, language and communication recovery**. While pharmacological and electromagnetic therapies show promise in consciousness recovery (including language function and communication), behavioral approaches involve sensory, language or music stimulation. BCIs and AAC provide tools for reestablishing and optimizing communication skills in DoC and EMCS patients, respectively. Created with Biorender.com.

A recent review identified five categories of treatments designed to promote recovery of consciousness in patients with severe brain injury: pharmacological therapies, electromagnetic therapies, mechanical therapies (e.g. low intensity focused ultrasound pulsation), sensory therapies (e.g. tactile and auditory stimulation), and regenerative therapies (e.g. stem cell, neurogenesis, gliogenesis, and axonal regrowth therapies) (Edlow et al., 2021). These treatment approaches illustrate the wide range of strategies that can be explored to support and enhance consciousness recovery, which aligns with the re-establishment of language and communicative functions. We describe those approaches that may be relevant to neuropsychological or speech-language therapies in the following sections (Figure 4).

### 4.1. Behavioral approaches

Multimodal stimulation approaches, including sensory therapies, have been employed for decades in the rehabilitation of patients with DoC (Giacino, 1996). These interventions involve stimulating various sensory modalities, with tactile and auditory stimuli being the most used. Examples of such programs include motor-based therapy, auditory training, music therapy, and multi-sensory stimulation (Thibaut, Schiff, et al., 2019). The underlying principle of these approaches is based on the idea that exposure to environmental stimuli can enhance neural processing, promote neuroplasticity, and support the recovery of consciousness. However, despite their widespread use, robust evidence supporting their efficacy remains limited, highlighting the need for further research in this area (Schnakers et al., 2016).

A double-blind randomized placebo-controlled trial examined the effects of Familiar Auditory Sensory Training (FAST) on neurobehavioral functioning, arousal, and awareness in 15 participants with DoC following severe traumatic brain injury (mean of 70 days post-injury) (Pape et al., 2015). The FAST protocol consists of 5-minute stories narrated by the patient’s relatives, focusing on autobiographical events. This was administered four times a day, with each session lasting 10 minutes and at least 2 hours between sessions, over a period of 6 weeks. The placebo protocol involved periods of silence. While both FAST and placebo groups demonstrated neurobehavioral improvements, the FAST group exhibited significantly greater gains in arousal and awareness and increased fMRI activation in language regions and whole-brain areas, similar to activation patterns seen in healthy subjects. These findings suggest that FAST may enhance neurorehabilitation outcomes and should be considered as a treatment option for DoC patients. A meta-analysis furthermore indicates that music therapy may improve functional outcomes for DoC patients, incorporating randomized controlled trials and cohort studies involving 201 patients (Li et al., 2020). This form of stimulation can be easily administered at the bedside. Additionally, exposure to preferred music has been shown to improve functional connectivity within the auditory network and attentional network of patients (Heine et al., 2015).

Language rehabilitation outcomes can vary significantly among DoC patients, requiring personalized interventions based on individual assessments and responsiveness. Repetition is often employed to establish habitual responses, while enhancing arousal and encouraging interactive communication is integrated throughout all therapies and interactions that patients may receive. Education and training for family members and caregivers are also crucial components of the rehabilitation process (Seel et al., 2013).

### 4.2. Pharmacological and electromagnetic therapies in DOC

Various pharmacological agents have been employed to enhance consciousness and support functional recovery in patients with DoC. They include amantadine, intrathecal baclofen, zolpidem, midazolam, and ziconotide. Among these pharmacological treatments, amantadine has been recommended for UWS and MCS patients (4–16 weeks after a traumatic brain injury) by the latest American practice guidelines based on one randomized controlled trial (Giacino et al., 2018). In addition, promising preliminary data were recently published on the potential therapeutic role for apomorphine (Sanz et al., 2024) and ketamine (Cardone et al., 2025) in DoC.

Non-pharmacological treatments, particularly electromagnetic therapies (both non-invasive and invasive), hold potential for promoting recovery of consciousness and improving language abilities in patients with DoC. Non-invasive techniques include transcranial direct current stimulation (tDCS) (Thibaut et al., 2023), repetitive transcranial magnetic stimulation (rTMS) (Vitello, Rosenfelder, et al., 2023), and transcutaneous auricular vagal nerve stimulation (taVNS) (Vitello, Briand, et al., 2023). Invasive methods, such as deep brain stimulation (DBS) (Chudy et al., 2023) and vagal nerve stimulation (VNS) (Dong et al., 2023), have also shown some potential as therapies for DoC. These approaches primarily modulate neural excitability at the cortical (i.e., via top-down mechanisms), central, or subcortical (i.e., via bottom-up mechanisms) levels. tDCS, when applied to the left dorsolateral prefrontal cortex, has been consistently shown to be a safe and feasible method for promoting recovery of consciousness. However, evidence suggests that it may only be beneficial for patients in a MCS (Thibaut, Bodien, et al., 2019; Thibaut et al., 2014). While other techniques have shown promise, additional research is needed to confirm their efficacy. In line with the association between language and consciousness recovery that is described above, investigating the effect of stimulations on language-specific brain areas could constitute another promising perspective.

### 4.3. Augmentative and alternative communication

For patients demonstrating communication skills (i.e., EMCS) but severely impaired motor output, the use of augmentative and alternative communication (AAC) should be considered as part of speech-language therapy management. High-tech AAC tools including those utilizing digital devices like tablets, speech-generating devices, and eye-tracking technology, have shown promising results in improving communicative skills in various neurological conditions (Formica et al., 2024). Studies have demonstrated their effectiveness in patients with post-stroke aphasia, LIS, amyotrophic lateral sclerosis, and traumatic brain injury. These tools not only enhance communication abilities but also positively impact social skills, anxiety levels, depression, and overall quality of life for patients (Formica et al., 2024). Low-tech AAC solutions should not be overlooked, as they can be equally valuable for some patients. These may include picture boards, communication books, or simple yes/no signals. The choice between high-tech and low-tech solutions should be based on the individual patient’s needs and cognitive abilities (Fried-Oken et al., 2015). Implementing AAC in the management of patients with DoC requires a collaborative approach. Speech-language therapists play a crucial role in assessing the patient’s communication needs, selecting appropriate AAC tools, and providing training to both the patient and their communication partners. It is finally essential to involve family members, caregivers, and other healthcare professionals in this process to ensure consistent and effective use of the chosen AAC system (Baxter et al., 2012; Mackey et al., 2023).

### 4.4. Brain-computer interfaces

BCIs provide alternative communication pathways by translating central nervous system activity into output, potentially enabling patients to interact with their environment (Wolpaw et al., 2020). While promising, these systems face challenges in patients with DoC due to the need for consistent and reproducible signals, and the requirement for patients to have preserved sensory and cognitive abilities to use the system effectively (Schiff et al., 2024). Furthermore, the type of BCI and the paradigm used seem to influence its effectiveness.

As described in section 3, there is a wide variety of sensorimotor, visual and auditory paradigms (Vatrano et al., 2023), and some underlying psychophysiological and neurological factors remain to be elucidated (Saha et al., 2021). The literature revealed several cases of patients behaviorally diagnosed with UWS who can communicate when evaluated by non-traditional techniques. For example, patients can be asked to mentally imagine situations (e.g., playing tennis or moving around his/her house) in an fMRI scanner, and then asked to use these situations to answer questions. In an fMRI study, a patient was able to correctly answer (5/6) autobiographical questions by mentally imagine playing tennis to say ‘yes’ and moving around his house to say ‘no’ (Monti et al., 2010). In another study based on EEG (Wang et al., 2017), the instruction given to patients was to focus on the ‘yes’ or ‘no’ presented on the screen to respond to the question that was asked, enabling to establish a communication code with one of them. The fNIRS technique could finally identify residual communication skills in one patient behaviorally diagnosed as UWS (Li et al., 2021).

The P300 wave, as observed during the presentation of a rare stimulus, is one of the most used EEG signals in BCI systems (Annen et al., 2020). When detected, this wave indicates that the patient has successfully identified the rare stimulus, enabling the detection of residual levels of consciousness (Chapman & Bragdon, 1964). Additionally, the P300 wave can be employed in BCI systems to assist patients in writing via a virtual keyboard. The principle is as follows: the patient focuses on a specific character they wish to select, and a P300 wave is elicited whenever the row or column containing the target character flashes (Ortner et al., 2011). Although this system is only suitable for patients with intact visual abilities, it enables free communication, extending beyond simple yes/no responses. The N400 wave detected in passive listening paradigms seems to be another interesting signal to use for BCIs. Indeed, this signal allows for the deduction of a person’s ‘mental context’, as it provides information on how individuals process and interpret presented stimuli (words or images) based on their current mental context (Dijkstra et al., 2020).

Although BCIs seem to be a promising therapeutic approach for enabling communication in this population, patients with CMD may be unable to use these techniques, just as some MCS+ patients may be unable to produce a reliable motor output to successfully engage with these systems (Claassen et al., 2023). It is therefore advised to adapt the BCI to each patient, considering his or her residual abilities and limitations, in order to select the most suitable paradigm for each individual (Galiotta et al., 2022). To obtain higher-quality data, a new approach to BCIs has finally been proposed: hybrid BCIs. These systems combine different signals or sensors, such as EEG, fMRI, electromyographic (EMG) signals, eye movements (EOG), visual stimuli (P300), and motor imagery, among others (Pfurtscheller, 2010). Several studies have demonstrated the effectiveness of this technique, including a BCI combining P300 wave detection with an eye-tracking system or visual evoked potentials to create a yes/no communication system. This approach achieved 100% accuracy in healthy subjects and showed promising results in two UWS patients (Huang et al., 2021; Li et al., 2022; Yi et al., 2024). An improvement in communication CRS-R subscale scores was also supported for two patients following the use of this BCI (Huang et al., 2021).

Research has additionally highlighted the benefits of neurofeedback, an innovative technique that allows individuals to monitor and modify their brain activity in real time using information about the brain’s response obtained through fNIRS, fMRI, or EEG (Klein et al., 2024). Notably, an fMRI study demonstrated an increase in the BOLD signal in brain regions associated with language processing—namely, the inferior frontal gyrus (Broca’s area) and the superior temporal gyrus (Wernicke’s area)—in patients with aphasia following neurofeedback training sessions (Sreedharan et al., 2020). Nevertheless, their improvement in language ability was not significantly greater than that of control patients with usual treatment. Progresses in the efficacy of this approach are therefore needed, and even more when targeting patients with DoC. The lack or inconsistency of responsiveness in these patients may indeed limit the efficacy of neurofeedback tools, which rely on their ability to respond to feedback and adjust their brain activity accordingly (Vatrano et al., 2023). The processing and analysis of neurofeedback signals also present unique challenges, including the inherent variability of brain activity in DoC conditions. Abnormal signals can mask or distort the signals of interest in neurofeedback applications, such as those associated with cognitive processing (Vatrano et al., 2023).

The use of BCIs in patients with DoC appears particularly promising, especially for communication reinstatement. These systems, however, remain hard to transpose in their daily life. Moreover, a recent hypothesis and theory paper raised awareness for including the detection of language abilities and language training of the BCI user as well as cultural differences as human factors in BCI research (Herbert, 2024). These gaps should be considered in future studies to allow for an adapted application of BCI technology in individuals with DoC.

Overall, rehabilitation requires multidisciplinary teams performing continuous assessment and tailoring strategies based on individual progress. There are multiple treatment options for consciousness recovery, such as pharmacological, electromagnetic, sensory or cognitive therapies. These strategies align with efforts to restore language and communication functions. For patients with communication abilities but limited motor function, high-tech and low-tech AAC tools help rehabilitate communication skills, while BCI techniques show promise in helping patients with DoC communicate. In summary, while significant advancements are being made in the treatment and rehabilitation of DoC patients, these therapies—ranging from behavioral interventions to advanced technologies—require more research and clinical trials to ensure their effectiveness.

## 5. Conclusion

This review emphasizes the difficulty in separating language, communication and consciousness impairments, at both diagnostic or rehabilitation levels, either behaviorally (e.g., when using standardized scales) or in neuroimaging and electrophysiological assessments.

All behavioral scales for diagnosing acute and chronic stages of DoC rely on verbal instructions that patients must comprehend to respond correctly. Additionally, the recovery of language and communication plays a key role in distinguishing between different levels of consciousness within the existing DoC classification system (Giacino et al., 2004). As a result, language impairments complicate the accurate assessment of consciousness (Schnakers et al., 2015). New tools specifically developed for assessing language and communication in DoC (Aubinet, Chatelle, et al., 2021; De Bellis et al., 2022) hold promise for better disentangling residual language and consciousness, which could enhance diagnostic accuracy and help tailor rehabilitation strategies.

Besides, most neuroimaging assessment techniques detecting covert consciousness include passive and active paradigms, both relying on language processing. Passive listening of linguistic stimuli revealed that some patients diagnosed with UWS exhibit brain activity related to higher-order language processing (e.g., Formisano et al., 2019; Kotchoubey, Yu, et al., 2013). These responses, detected via fMRI or EEG, were associated with better recovery outcomes, although they are often weaker and less extensive than those observed in MCS patients. Active paradigms, which require intentional responses to verbal commands, have identified CMD in 25% of patients, demonstrating residual cognitive abilities that behavioral assessments alone fail to detect (Bodien et al., 2024). However, factors such as aphasia or methodological limitations can complicate the interpretation of these findings (Jacobson et al., 2024).

Similarly, different therapeutic options dedicated to the recovery of consciousness are also related to the reappearance of language comprehension and communication abilities. Auditory training such as the FAST (and music therapy to a lesser extent) includes speech processing and showed improvements regarding consciousness recovery (Pape et al., 2015). Electromagnetic therapies such as tDCS, rTMS, and taVNS have shown potential to support recovery of consciousness and language function, though more research is needed to confirm efficacy (Thibaut, Schiff, et al., 2019). BCI systems further involve techniques like EEG, fMRI, or neurofeedback. Despite challenges, they have demonstrated success in some patients, enabling communication through methods like mental imagery or eye-tracking. Hybrid BCIs, combining multiple signals, show even more promise (Pfurtscheller, 2010).

The Curing Coma Campaign recently convened work groups to address clinical and technical challenges related to CMD (Claassen et al., 2023) and the use of BCIs for communication in patients with DoC (Schiff et al., 2024). Among other points, these groups emphasize the uncertainty surrounding the underlying mechanisms of CMD, the methodological variability that limits its utility as a biomarker for prognosis and therapeutic trials, the difficulty in identifying patients with covert consciousness who might be capable of communication via BCIs, and the complex pathophysiological effects of heterogeneous brain injuries on neuronal signaling. This heterogeneity underscores the need for personalized BCIs to ensure their successful translation into clinical settings (Schiff et al., 2024). Investments in research, infrastructure, and education are also crucial for the clinical implementation of these techniques, as their use requires highly trained and specialized personnel (Claassen et al., 2023; Young et al., 2025).

Combining multimodal approaches aimed at more accurate language and consciousness assessments, including behavioral scales and both passive and active tasks, paves the way for improved consciousness evaluations and targeted interventions to support communication and recovery in patients. Future research should focus on refining existing tools, developing standardized protocols, and exploring innovative therapies that incorporate language recovery and communication support. Their practical use in everyday life remains a challenge, necessitating further research on personalized approaches. Clinicians and researchers would therefore be able to enhance the quality of care, improve outcomes, and deepen our understanding of consciousness in patients with severe brain injury.
